# Supplementary material for: The characteristics and clinical course of patients with melioidosis and cancer
Source: PLoS Negl Trop Dis. 2024 Oct 25;18(10):e0012631. doi: 10.1371/journal.pntd.0012631 (PMC11540213; doi:10.1371/journal.pntd.0012631)
Supplement: S1 Table — (DOCX) [file pntd.0012631.s001.docx]

**Supplementary table 1.** Definitions of the different clinical phenotypes used in the study

**Lung involvement**: growth of *B. pseudomallei* in sputum or from another site (blood, urine, or other body fluid) with concomitant radiological imaging suggesting pulmonary involvement.

**Genitourinary infection**: growth of *B. pseudomallei* in urine or from another site (blood, sputum, or other body fluid) with concomitant radiological imaging suggesting genitourinary involvement.

**Musculoskeletal involvement**: defined as a pyomyositis, septic arthritis or osteomyelitis confirmed by culture of *B*. *pseudomallei* from an aspirate of an abscess in muscle, or joint fluid or bone culture. Alternatively imaging or intra-operative findings consistent with pyomyositis, septic arthritis or osteomyelitis with *B*. *pseudomallei* isolated concurrently from a microbiological sample from elsewhere in the body.

**Skin or soft tissue infection**: growth of *B. pseudomallei* from a skin swab, or new clinical findings consistent with skin involvement with *B*. *pseudomallei* isolated concurrently from a microbiological sample from elsewhere in the body.

**Central nervous system involvement**: Radiological changes or laboratory investigations consistent with active central nervous system infection (encephalomyelitis, a brain abscess, meningitis, or extra-meningeal involvement) and a positive *B. pseudomallei* culture from a microbiological sample from the central nervous system or from elsewhere in the body.

**Bacteraemia**: Isolation of *B. pseudomallei* from blood culture.
