# Supplementary material for: The characteristics and clinical course of patients with melioidosis and cancer
Source: PLoS Negl Trop Dis. 2024 Oct 25;18(10):e0012631. doi: 10.1371/journal.pntd.0012631 (PMC11540213; doi:10.1371/journal.pntd.0012631)
Supplement: S2 Table — (DOCX) [file pntd.0012631.s002.docx]

**Supplementary table 2.** Comparison of the characteristics of the individuals with a solid organ tumour and those with a haematological malignancy.

|  | All n = 446 ^a^ | Solid organ tumour n=36 | Haematological malignancy n = 11 | Odds ratio (95% confidence interval) | p |
| --- | --- | --- | --- | --- | --- |
| Age (years) | 55 (43-66) | 67 (57-73) | 67 (58-78) | 0.98 (0.92-1.04) | 0.58 |
| Male sex | 313 (70%) | 27 (75%) | 8 (73%) | 1.13 (0.24-5.18) | 0.88 |
| First Nations Australian | 207 (46%) | 9 (25%) | 0 | - | - |
| Remote residence ^b^ | 150 (33%) | 6 (17%) | 2 (18%) | 0.90 (0.15-5.26) | 0.91 |
| Wet season presentation | 332 (74%) | 25 (69%) | 9 (82%) | 0.51 (0.09-2.73) | 0.43 |
| Diabetes mellitus | 226 (51%) | 8 (22%) | 3 (27%) | 0.76 (0.16-3.56) | 0.73 |
| Hazardous alcohol use | 170/440 (39%) | 8/35 (23%) | 2 (18%) | 1.33 (0.24-7.47) | 0.74 |
| Tobacco smoker | 220/440 (50%) | 18/35 (51%) | 2 (18%) | 4.76 (0.90-25.3) | 0.07 |
| Chronic lung disease | 95 (21%) | 14 (39%) | 3 (27%) | 1.70 (0.38-7.50) | 0.49 |
| Chronic kidney disease | 55 (12%) | 4 (11%) | 0 | - | - |
| Immunosuppression | 58/327 (18%) | 21 (58%) | 7 (63%) | 0.80 (0.20-3.23) | 0.75 |
| Lung involvement | 325/444 (73%) | 28 (78%) | 7 (64%) | 2.00 (0.47-8.60) | 0.35 |
| Genitourinary involvement | 88/439 (20%) | 6 (17%) | 0 | - | - |
| Musculoskeletal involvement | 55/438 (13%) | 2 (6%) | 1 (9%) | 0.59 (0.05-7.18) | 0.68 |
| SSTI | 63/438 (14%) | 1 (3%) | 1 (9%) | 0.29 (0.02-4.99) | 0.39 |
| CNS involvement | 16/438 (4%) | 0 | 0 | - | - |
| Bacteremia | 314 (70%) | 29 (81%) | 9 (82%) | 0.92 (0.16-5.25) | 0.93 |
| Septic shock | 91/428 (21%) | 8/35 (23%) | 0 | - | - |
| ICU admission | 107 (24%) | 7 (19%) | 0 | - | - |
| Died before discharge from melioidosis | 40 (9%) | 6 (17%) | 1 (9%) | 2.00 (0.21-18.69) | 0.54 |

SSTI: Skin and soft tissue infection. CNS: Central nervous system. ICU: Intensive Care Unit.

^a^ It was possible to reliably confirm or exclude an active cancer in only 446/477 patients in the cohort. Retrospective data collection from cases before October 2016 resulted in some missing data prior to this time and, accordingly, a difference in the denominator for some variables.

^b^ Patients living in the Torres and Cape Hospital and Health Service
