# Supplementary material for: The characteristics and clinical course of patients with melioidosis and cancer
Source: PLoS Negl Trop Dis. 2024 Oct 25;18(10):e0012631. doi: 10.1371/journal.pntd.0012631 (PMC11540213; doi:10.1371/journal.pntd.0012631)
Supplement: S3 Table — (DOCX) [file pntd.0012631.s003.docx]

**S3 Table.** Comparison of the characteristics of the patients who had no cancer therapy prior to the diagnosis of melioidosis and the patients that did (stratified by whether they did – or did not – take TMP/SMX prophylaxis)

|  | No anti-cancer therapy in the 12 months prior to diagnosis of melioidosis n=30 | Received TMP/SMX prophylaxis during anti-cancer therapy in the 12 months prior to diagnosis of melioidosis ^a^  n=1 | Did not receive TMP/SMX prophylaxis during anti-cancer therapy in the 12 months prior to diagnosis of melioidosis ^a^ n=16 |
| --- | --- | --- | --- |
| Age (years) | 67 (56-75) | 64 | 62 (49-67) |
| Male sex | 25 (83%) | 1 (100%) | 9 (56%) |
| First Nations Australians | 7 (23%) | 0 (0%) | 2 (13%) |
| Remote residence ^b^ | 7 (23%) | 0 (0%) | 1 (6%) |
| Wet season presentation | 21 (70%) | 1 (100%) | 12 (75%) |
| Diabetes mellitus | 9 (30%) | 0 (0%) | 2 (13%) |
| Hazardous alcohol use | 9/29 (31%) | 0 (0%) | 1 (6%) |
| Smoker | 12 (40%) | 0 (0%) | 8 (50%) |
| Chronic lung disease | 12 (40%) | 0 (0%) | 5 (31%) |
| Chronic kidney disease | 4 (13%) | 0 (0%) | 0 (0%) |
| Immunosuppression | 12 (40%) | 1 (100%) | 15 (94%) |
| No other risk factors for melioidosis | 10 (33%) | 1 (0%) | 9 (56%) |
| Lung involvement | 21 (30%) | 1 (100%) | 13 (81%) |
| Genitourinary involvement | 4 (13%) | 0 (0%) | 2 (13%) |
| Musculoskeletal involvement | 2 (7%) | 0 (0%) | 1 (6%) |
| SSTI | 2 (7%) | 0 (0%) | 0 |
| CNS involvement | 0 (0%) | 0 (0%) | 0 (0%) |
| Bacteraemia | 22 (73%) | 1 (100%) | 15 (94%) |
| Septic shock | 7/29 (24%) | 0 (0%) | 1 (6%) |
| ICU admission | 7 (23%) | 0 (0%) | 0 |
| Died from melioidosis before hospital discharge | 4 (13%) | 0 (0%) | 3 (19%) |

^a^ TMP-SMX prophylaxis for *Pneumocystis jirovecii* pneumonia as per the anti-cancer therapy protocol (160/800mg twice a day on Monday and Thursday)

^b^ Patients living in the Torres and Cape Hospital and Health Service
