# Supplementary material for: The characteristics and clinical course of patients with melioidosis and cancer
Source: PLoS Negl Trop Dis. 2024 Oct 25;18(10):e0012631. doi: 10.1371/journal.pntd.0012631 (PMC11540213; doi:10.1371/journal.pntd.0012631)
Supplement: Attachment — Submitted filename: PNTD-D-24-00958_ Response to reviewers.docx [file pntd.0012631.s004.docx]

**Response to reviewers**

We thank the Editorial staff and the three reviewers for the time that they have taken to review our manuscript and the very helpful comments that they have made to improve the work. Please find below our point-by-point responses (in black) to their comments (in blue).

We note that there was significant disparity in the reviewers’ comments: reviewers 1 and 2 were very positive and had few suggestions for the paper’s enhancement. Reviewer 3 was less positive; we have done our best to address his/her concerns in the revised manuscript.

Editorial comment:

Reviewer 3 raises multiple concerns relating to methodology, analysis, ethics, and presentation/interpretation. Please carefully address each of these concerns in your revision.
**Response**: While Reviewers 1 and 2 appeared to enjoy reading the original manuscript and had no – or very few – concerns about the methodology, analysis, ethics, and presentation/interpretation, we agree Reviewer 3 has raised multiple concerns. Please see our point-by-point responses below.

Reviewer's Responses to Questions

Key Review Criteria Required for Acceptance?
As you describe the new analyses required for acceptance, please consider the following:

Methods
-Are the objectives of the study clearly articulated with a clear testable hypothesis stated?
-Is the study design appropriate to address the stated objectives?
-Is the population clearly described and appropriate for the hypothesis being tested?
-Is the sample size sufficient to ensure adequate power to address the hypothesis being tested?
-Were correct statistical analysis used to support conclusions?
-Are there concerns about ethical or regulatory requirements being met?

Reviewer #1: Yes
**Response**: We thank Reviewer 1 for reviewing our paper and are pleased that they are happy with the methods.

Reviewer #2: Yes, method and statistical analysis used are appropriate.
**Response**: We thank Dr Chee Yik Chang for reviewing our paper and are pleased that he is also happy with the methods and statistical analysis.

Reviewer #3: 1. No hypothesis is provided for the study (usually in the introduction section but is a component of study methods).

**Response**: We did not use the word hypothesis, but the aims of the study are clearly explained in lines 149-153 of the introduction. We have adjusted the phrasing of this section to highlight the fact that there were two distinct components to the study (the reviewer appears, in other comments, not to have comprehended this completely): “This study had two separate aims. The first was to describe the burden, clinical characteristics, comorbidities, and outcomes of melioidosis in patients with cancer to define the clinical phenotype of melioidosis in this population more precisely. The second aim was to define the risk and benefits of TMP-SMX chemoprophylaxis for melioidosis in patients with cancer receiving chemotherapy to determine if this strategy should be adopted in the FNQ region.”

2. The study design is confusing as they state that it is prospective and retrospective.  It appears as though hospital databases are used for the study which then makes this fully retrospective. If it was prospective, I do not see that patients were approached for consent as they would be individual admissions. If the data was collected for example after someone's admission was done, that still makes it retrospective.

**Response**: We thank the reviewer for raising this point. What we were hoping to relate was that individuals with culture-confirmed melioidosis were identified and all relevant data were added to a dedicated database prospectively from October 2016. These data were collected and entered into the database retrospectively prior to this date (for the paper that we published in PLoS NTD in 2017 that examined all cases of melioidosis in the FNQ region between January 1, 1998 and July 31, 2016 [doi.org/10.1371/journal.pntd.0005411](https://doi.org/10.1371/journal.pntd.0005411)).

We highlighted this fact to emphasise that the data that we have collected on the patients with melioidosis is complete after October 2016, but there are some missing data for the patients that we identified retrospectively (a point we highlight in a footnote in table 1). We have amended the text to emphasise that the data were collated from the patients’ medical records and entered into a dedicated database (lines 170-171).

The process that we used was very similar to the world’s largest prospectively collected dataset of the melioidosis, the Darwin Prospective Melioidosis Study (doi.org/10.1016/ S1473-3099(21)00022-0), so we feel that researchers in the field will be familiar with the process.

As we highlight in the ethics section, as the data presented is de-identified, the FNQ Human Research Ethics Committee waived the requirement for informed consent (lines 222-223).

3. The databases and variables collected for the study should be described in one paragraph/section to make it clear what data was obtained from where/how and any definitions that may be relevant for socio-demographic and clinical variables. I think the clinical diagnosis and definitions of melioidosis does need to be described here like phenotypes/syndromes as it is relevant to the study itself and at minimum be included in a supplement.

**Response**: We thank the reviewer for raising this point. The data that were obtained are presented in the results; we are not sure that listing them all individually in the methods section adds much to the paper. We have highlighted that the precise definitions of the comorbidities that we used for this paper have been presented previously (lines 176-177, ref 20)

As described, all included patients had culture-confirmed melioidosis; that was our definition (lines 168-170). No patients were diagnosed “clinically”. We have added a supplementary table to define the clinical phenotypes as requested (S Table 1).

4. Where was the data compiled prior to de-identification? Were the identifying variables stored anywhere as a master dataset for linking?

**Response**: We thank the reviewer for raising this point. Data were collected from the patients’ (paper and subsequently electronic) medical records. These data are de-identified and entered into a dedicated database that is stored securely at Cairns Hospital in accordance with published guidelines from the National Health and Medical Research Council, Australian Research Council and Universities Australia; we have added a reference to highlight this (reference 22). The responsible management of these data is a requirement of the study’s ongoing ethics and governance approval, which was first granted in 2015, and which has been reviewed – and approved – annually since that time.

5. Greater detail on what variables were evaluated for the regression models should be included. What was the primary and/or secondary outcome(s)? Trends over time for what?

**Response**: We thank the reviewer for raising this point. We have added text to the statistical analysis section to address the reviewer’s concerns (lines 210-212 & 217-219)

6. For the incidence rates, what was the denominator used? How well does this dataset represent the population in this region? As there are two health systems, are they both within the region and if yes, would they not be added to get the population estimate for the FNQ?

**Response**: We thank the reviewer for raising this point. The study was performed in Far North Queensland in tropical Australia. The region contains two health services. We have amended the text to make this clearer (lines 163-166).

We did present the incidence data for the whole region in 2022 in the introduction (line 140), but we thought is prudent to present the incidence data for the two different health services separately as urban expansion has resulted in the incidence of melioidosis rising significantly in the CHHHS (where the population is older, and therefore at higher risk of malignancy). In contrast, the incidence in the TCHHS has been stable.

--------------------
Results
-Does the analysis presented match the analysis plan?
-Are the results clearly and completely presented?
-Are the figures (Tables, Images) of sufficient quality for clarity?

Reviewer #1: Yes

**Response**: We thank Reviewer 1, again, for reviewing our paper and are pleased that they are happy with the results.

Reviewer #2: Yes, clearly presented in the tables.

**Response**: We thank Dr Chee Yik Chang, again, for reviewing our paper and are pleased that he is also happy that the results are presented clearly in the tables.

Reviewer #3: 1. The p-value for table 1 should be to one decimal place and afterwards to two (with the exception of <0.001).

**Response**: We are not sure we follow Reviewer 3 here. It is customary to report a p value to 2 decimal places (apart from those with a p<0.001), and we have done this in all our prior PLoS NTD publications. Otherwise, both a p value of 0.06 and 0.14 would be presented as 0.1. We feel that this lacks the nuance that a reported p value of 0.06 and 0.14 can provide in a modestly sized sample.

We have presented the p value for SSTI to three decimal places (p=0.053) in table 1 to emphasise that the inverse association between SSTI and cancer was not statistically significant. The odds ratio (95% confidence interval) in the previous cell is 0.24 (0.06-1.02) and we feel that is important to emphasise that this was not a significant inverse association. This might be ambiguous for the casual reader if we just presented to 2 decimal places (i.e. p=0.05).

We have therefore not made any changes to the presentation of the p values in table 1. We apologise if we have misunderstood Reviewer 3’s point, although we note that neither Reviewer 1 nor Reviewer 2 expressed any concerns about the presentation of the data.

2. Were those who did not have ability to determine if there was cancer (~31) similar demographically to the cohort?

**Response**: We thank Reviewer 3 for raising this point. There were 31 patients in whom it was not possible to determine whether there was an active cancer at the time their melioidosis was diagnosed. All but one of these patients presented before data were collected prospectively; the one patient who was diagnosed with melioidosis after October 2016 was diagnosed post-mortem after being found dead in the community.

The other 30 patients were usually from early in the study period (17/30 were in the first 5 years of the study period). We were unable to determine whether they had an active cancer as many of these patients had had their paper charts destroyed, and an electronic medical record was not used in the health service until 2015.

Patients with missing cancer data were more likely to live remotely (21/31 (68%) versus 152/446 (34%), p<0.0001), were more likely to identify as First Nations Australians (25/31 (81%) versus 207/446 (46%), p<0.0001) and were more likely to be younger (median (IQR) age: 47 (33-59) versus 55 (43-66) years, p=0.02) than individuals who did have the presence or absence of cancer documented. This is in keeping which the changing epidemiology of melioidosis in the region (rising incidence in Cairns during the study period where the population is older and less likely to identify as a Fierst Nations Australians (a point we make in lines 335-338).

It is likely that, as the patients with missing data were more likely to be younger, to identify as First Nations Australians and to live remotely (all three factors associated with a lower rate of cancer in the patients in the cohort with complete data), they were also less likely to have cancer, but we have not made this assumption. As this does not change the findings of our paper, we have not added this data to the paper, although this will be available in the peer review history which we plan to make publicly available. We would be happy to add it to the paper if the Editor feels that it enhances the manuscript.

3. Is there an issue with the institutional ethics board with listing numbers less than 10 in Table 1? This can increase likelihood of having someone be identifiable and should be considered. I appreciate each person is described in a later table so as long as there are no concerns from the ethics board, that is okay.

**Response**: We thank the reviewer for raising this point; it is clearly important to maintain patient confidentiality. We have published >20 papers from this cohort since 2017 and they have all been provided to the Far North Queensland Ethics Committee as a part of the regular annual reporting process. Several of these papers have included tables with individual, de-identified case descriptions

doi: 10.1371/journal.pntd.0012317

doi: 10.1016/j.actatropica.2024.107209

doi: 10.4269/ajtmh.23-0350

doi: 10.1093/cid/ciac111

The Committee have expressed no concerns.

4. Table 1 has odds ratios and typically is meant to be descriptive of the cohort. If this is reflecting the regression, perhaps it is best put as a separate table to make clear what the regression is looking at and the results.

**Response**: We thank the reviewer for raising this point. Table 1 does present the characteristics of the overall cohort. The second column “All” describes this. The next two columns define the characteristics of the patients with – and without – a cancer and the fourth column presents the odds ratio (95% CI) in univariate analysis. We have described in the statistics section how we selected the variables for the multivariate analysis (lines 212-214), and we have identified the variables in the table (with a superscript d) which of the variables were selected for this multivariate analysis (the results of which are presented in the text (lines 238-241). It is, in general, our practice to not duplicate presentation of data in text and tables. We have added a superscript to the odds ratio column to highlight that this was only the univariate analysis.

We apologise if we have misunderstood the point that Reviewer 3 was making; we note that Reviewer 1 and 2 expressed no concerns about the tables.

5. Suggest changing the title to Association of TMP-SMX.

**Response**: We agree that the subheading “Benefit of TMP-SMX for prophylaxis of melioidosis” is imperfect. But we don’t feel that “Association of TMP-SMX” is necessarily better. It doesn’t say what the TMP-SMX is associated with. And really the aim of the study was to determine whether the prescription of TMP-SMX had an INVERSE association with the subsequent development of melioidosis. We have therefore changed the subheading to “Impact of TMP-SMX prophylaxis on the subsequent incidence of melioidosis”

--------------------

Conclusions
-Are the conclusions supported by the data presented?
-Are the limitations of analysis clearly described?
-Do the authors discuss how these data can be helpful to advance our understanding of the topic under study?
-Is public health relevance addressed?

Reviewer #1: Yes

**Response**: We thank Reviewer 1, again, for reviewing our paper and are pleased that they are happy with the conclusions.

Reviewer #2: Yes, the findings from this study further support active cancers and the treatment (chemotherapy) as significant risk factors for melioidosis. Bactrim prophylaxis to prevent melioidosis need to be further investigated to identify the high risk patients who may benefit from prophylaxis.

**Response**: We thank Dr Chee Yik Chang, again, for reviewing our paper and are pleased that he is also happy with the conclusions.

Reviewer #3: 1. In my opinion, the discussion is overstated. For example, they report NNT but cannot be certain that TMP-SMX is the only factor that 'prevented' an infection in the study. Given that only 11% of the cohort has a malignancy and that too highly variable in terms of type, duration and treatment, they are very underpowered to conclude that TMP-SMX is not helpful. While this may be true, the messaging needs to be changed and attenuated based on this study.

**Response**: We are concerned that Reviewer 3 may have misunderstood the design of the study. The NNT is not derived from the 446 patients in cohort with confirmed melioidosis, only 47 (11%) of whom had an active cancer. Rather it is derived from the 4536 patients with a cancer who received anti-cancer therapy at the hospital since 2007.

We have adjusted the presentation of the aims and the structure of the methods to highlight this and limit confusion.

There are actually two separate parts to the study.

The first examines all cases of culture proven melioidosis at our centre between January 1, 1998, and June 1, 2023. It examines, as we highlight in our introduction, the burden, clinical characteristics, comorbidities, and outcomes of melioidosis in patients. The aim is to define the clinical phenotype of melioidosis in patients with a concurrent cancer diagnosis more precisely (lines 149-151). The results of this part of the study are presented in lines 237-304 and tables 1,2 and 3. There were 477 cases of culture-confirmed melioidosis during the study period; in 446 it was possible to determine the presence – or absence – of a concomitant active cancer. There were 47/446 (10%) who had a concomitant cancer.

The second part of the study examined all individuals receiving anti-cancer therapy at our centre data after January 1, 2007. This part of the study aimed to define the risk and benefits of TMP-SMX chemoprophylaxis for melioidosis in patients with cancer receiving chemotherapy to determine if this strategy should be adopted in the FNQ region (lines 151-153). The results of this part of the study are presented in lines 305-314 and table 4. Between 1 January 2007 and June 1, 2023, there were 4088 patients with cancer who received myelosuppressive chemotherapy and 448 individuals with cancer who received non-immunosuppressive anti-cancer therapy at our centre. We examined the incidence of melioidosis in these 4536 patients with cancer, 652 of whom received TMP-SMX as part of the anti-cancer therapy regimen (in these protocols this is primarily to reduce the risk of *Pneumocystis jirovecii* infection).

While 11% of the patients with melioidosis had an active cancer, we did not examine TMP-SMX prophylaxis in these patients (very few of whom were receiving it). We examined the efficacy of TMP-SMX prophylaxis in the 4536 patients (who all had a cancer) receiving anti-cancer therapy. We would argue that the prescription of TMP-SMX in 652 of 4536 patients would mean that this sample is not “very underpowered” to answer our study question.

Table 4 presents the number of patients receiving TMP-SMX as part of their anti-cancer therapy regimen, stratified by intensity of the regimen. This TMP-SMX was prescribed to prevent *Pneumocystis jirovecii* not melioidosis (as we state in lines 204-205). These data were then be used to determine if TMP-SMX has any utility in the prevention of melioidosis. The proportion of confirmed cases of melioidosis in the patients receiving – and not receiving – TMP-SMX prophylaxis was used to (crudely) determine the number needed to treat (NNT) to prevent one case of melioidosis.

The reviewer states that, “they report NNT but cannot be certain that TMP-SMX is the only factor that 'prevented' an infection in the study”, this is true, but the data that we present gives the reader some idea of the absolute risk of melioidosis in all patients receiving anti-cancer therapy and the absolute risk reduction of melioidosis in the patients receiving TMP-SMX at different intensities of myelosuppression.

The reviewer is right to state that other factors influence the risk of melioidosis (predisposing comorbidities and environmental exposure) and we were unable to define these data in the 4856 patients receiving chemotherapy. We have added some text to the limitations to state this explicitly (lines 425-428), however, we feel that we have demonstrated clearly that the risk of developing melioidosis during anti-cancer therapies in this region of Australia is very low and there is likely to be little incremental benefit in adding prophylactic TMP-SMX to the treatment regimens of all people in this region of tropical Australia receiving chemotherapy to prevent melioidosis, as is recommended in current Australian guidelines (lines 123-130, ref 13).

2. The changing incidence could be better explored by looking at data outside of their study cohort and the risk factors. Has this changing incidence been seen in other parts of australia or other countries with a high incidence?

**Response**: We thank the reviewer for raising this point. The dramatic change in local incidence of melioidosis has been closely linked to recent urban expansion south of Cairns as we state in lines 335-338. We provide a reference for this statement (reference 25), a paper which explored the association between this urban expansion and the local incidence of melioidosis cases in some detail.

Environmental disturbance has been linked to changes in the *B. pseudomallei* in other parts of Australia as we highlight in the introduction (reference 3). Other factors that might be liked to a changing incidence of melioidosis include changes in the burden of comorbidities that predispose to melioidosis (such as the increasing global incidence of diabetes and, relevant to this paper, cancer) and even climate change (references 1 and 5).

However, the tenfold rise in melioidosis incidence occurred in the Cairns region over a very short period of time. We would not expect to see such a rapid change (>10 fold) in incidence due to local changes in the prevalence of comorbidities (which have not changed by this degree in the same time period, table 2, reference 25) or due to climate change (which we have examined in reference 4 showing no link between changes in incidence and climactic factors), Furthermore if comorbidity or climate factors were felt to be contributing to such impressive changes in incidence, we would not expect to see such a stable incidence in neighbouring parts of FNQ.

Changes in the burden of comorbidities and an increase in sever weather events – and improvements in diagnostic capacity – may contribute to a rising incidence of melioidosis in other parts of the world, but we feel that more discussion of this subject this is beyond the scope of this paper which has a focus on clinical practice in FNQ, Australia.

3. The statement of alcohol and smoking is not that relevant to this study and therefore should be again re-stated vs applying it to this cohort. Were there any other epidemiologic exposures that were considered?

**Response**: We respectfully disagree with the reviewer on this point. Smoking and alcohol are two of the 5 strongest risk factors for cancer in Australia: https://www.canceraustralia.gov.au/resources/position-statements/lifestyle-risk-factors-and-primary-prevention-cancer/lifestyle-risk-factors

They also increase the risk of melioidosis, either directly in the case of alcohol (in the Darwin Prospective Melioidosis Study it was the second most common predisposing factor for melioidosis, present in 40% of the patients a figure similar to the 39% seen in our cohort) or indirectly in the case of cigarette smoking (in the Darwin Prospective Melioidosis Study chronic lung disease was the third most common predisposing factor for melioidosis, present in 27%% of the patients a figure similar to the 21% seen in our cohort.

It was therefore somewhat surprising to us – and we believe noteworthy and worth highlighting – that the patients with melioidosis and cancer in our cohort were not more likely to be smoking or drinking hazardously than patients without cancer. However, as we suggest, this may reflect changes in lifestyle precipitated by their diagnosis of cancer and its treatment (lines 347-349)

4. I think much of the septra and immunosuppressive discussion can be reduced as this was not the specific goals of their study (beyond looking at septra and melioid risk/outcomes).

**Response**: We respectfully disagree with the reviewer on this point. The focus on TMP-SMX (trade name Septra) was actually one of the two stated – or “specific” – aims of the study. We note that reviewer 1 stated that “the discussion regarding prophylactic use of TMP-SMX was excellent.”

5. The limitations paragaph needs to be improved. How did these limitations affect the study results and/or the interpretation of the study? What was done to mitigate these effects?

**Response**: The reviewer has not specifically stated which additional limitations we need to highlight. We fear that he/she may have misunderstood the design of the study, and this may have led them to believe that there were additional limitations. We have done our best to add some limitations to this section based on Reviewer 3’s comments above (lines 418-419, 425-428)

--------------------

Editorial and Data Presentation Modifications?
Use  this section for editorial suggestions as well as relatively minor modifications of existing data that would enhance clarity. If the only modifications needed are minor and/or editorial, you may wish to recommend “Minor Revision” or “Accept”.

Reviewer #1: (No Response)

Reviewer #2: Accept
**Response**: We thank Dr Chee Yik Chang, again, for reviewing our paper and are encouraged that he has recommended the paper be accepted.

Reviewer #3: (No Response)

--------------------
Summary and General Comments
Use  this section to provide overall comments, discuss strengths/weaknesses of the study, novelty, significance, general execution and scholarship. You may also include additional comments for the author, including concerns about dual publication, research ethics, or publication ethics. If requesting major revision, please articulate the new experiments that are needed.

Reviewer #1: I enjoyed reading this manuscript.
The methodology is sound, the results are well presented, and the discussion regarding prophylactic use of TMP-SMX was excellent.

**Response**: We thank Reviewer 1, again, for reviewing the paper. We are very encouraged by their very positive feedback.

3 questions:
1.      Why did the authors use “died before discharge from melioidosis” (which could be phrased "died from melioidosis before discharge"), and not the total deaths from melioidosis irrespective of discharge?

**Response**: We thank Reviewer 1 for raising this point. We have revised table 1 to use “died from melioidosis before discharge” as suggested. Death before discharge seemed a logical endpoint for us as every patient in the cohort had been discharged (and therefore had an outcome). As we included all patients with culture-confirmed melioidosis until 1 June 2023, some have had < 2 years of follow up. There is the possibility that some of these patients will have relapse of their melioidosis and there is the possibility that they will die from this relapse. We do highlight the one additional death from melioidosis in the 47-year male with hepatocellular carcinoma who died with presumed relapse of melioidosis (clinical diagnosis, not culture confirmed). That being said, relapse is a relatively rare occurrence at our centre and so it would not be expected to make any meaningful difference to our findings.

2.      Is there a reference for the myelosuppressive grading scale used? If not, how confident are the authors of the accuracy of these subpopulations?

**Response**: We thank the reviewer for raising this point. The myelosuppressive potential of each of the chemotherapy regimens was determined using National Comprehensive Cancer Network guidelines. We have rephrased the text to highlight this (line 197) and added a reference (ref 21).

3.      Partially out of interest, but with relevance to the paper, do the authors know the expected 12-month mortality for cancer patients with non-melioidosis infections in their region?

**Response**: The reviewer raises a good point. We do not have this data, but it would be difficult to present a highly heterogenous dataset cohesively and there are many factors that would influence this.

Some cancers have an intrinsically poorer prognosis that others (e.g. cholangiocarcinoma versus prostate cancer). Cancers may be metastatic or non-metastatic (prostate disease is another good example here). Cancers can be primary or relapsed (and hence more intrinsically resistant to therapy). There may be differences in treatment (bone marrow transplant, high risk chemotherapy versus watching and waiting). There are patient factors (age and comorbidities, access to care) and finally there are pathogen related factors (melioidosis has a far higher case fatality rate than, say, skin and soft tissue infections). We agree that this is an interesting point, but it is probably beyond the scope of this report that focuses on melioidosis.

a.      Similarly, is the 61% non-melioidosis-associated mortality expected or surprisingly high?

**Response**: We thank the reviewer for raising this point. We agree that the 61% mortality for non-melioidosis death is, on the face of it, a high figure. However, examining the data more closely it may be explicable. There were 13 deaths from cancer in the 12 months after the melioidosis diagnosis, but the majority (7/13 (54%)) were in patients with metastatic disease, which in itself has a poor prognosis. Of the remaining 6 patients with non-metastatic disease 5 had cancers with a poor prognosis: 1 had cholangiocarcinoma (median survival from diagnosis 6-12 months), 1 had HCC (median 12 months survival 40%), 1 had oesophageal cancer (median 5-year survival 23%), 1 had two cancers (a supraglottic SCC and a synchronous lung cancer, both cancers with a poor prognosis), 1 had diffuse B cell lymphoma, which had a poor prognosis as the patient had had a prior autologous stem cell transplant and was on multiagent chemotherapy and high dose corticosteroids, while the final patient had significant myelodysplasia (with pancytopenia). The cachexia/frailty occasioned by the metastatic/aggressive cancer may have both increased their risk for melioidosis influenced their prognosis. We have added some text to highlight this finding (lines 367-370)

Reviewer #2: This is an important paper.

**Response**: We thank Dr Chee Yik Chang, again, for reviewing our paper and are heartened that he feels that this is important work.

Reviewer #3: The authors present the results of a retrospective cohort study comparing outcomes of melioidosis in those with and without cancer. They identified that cancer of various types was present in 11% of cases and that there were some differences in characteristics. Those who received TMP were slightly less likely to develop infection but outcomes (death) were not significant different.

The methods are unclear at times and the analysis approaches should be made clearer.

**Response**: We are uncertain if the Reviewer has understood all the elements of our paper correctly, however we have amended the paper to, hopefully, make several points clearer, particularly the structure of the methods to emphasise that the paper examines two – almost mutually exclusive – cohorts.

The cohort is highly heterogeneous as it relates to cancer based on type, time (~2 decades), treatment, duration, stage etc and the impact of this on the study is not well considered.

**Response**: We agree with the reviewer. Indeed, in the initial submission we highlighted this as a specific limitation of the study, “The patients with a malignancy were a highly heterogeneous population ‒ there is a significant difference between a patient with diffuse large B-cell lymphoma and a previous stem cell transplant receiving chemotherapy and a patient with localised prostate cancer who is managed with watchful waiting.” (lines 419-423). We have added some text about the evolution of treatment of melioidosis and cancer over the study period (lines 418-419).

The study is underpowered and this is not acknowledged by the investigators. Thus, much of the messaging in the discussion and conclusion needs to be tempered with this in mind.

**Response**: As we have highlighted above, we feel that Reviewer 3 has fundamentally misunderstood the design of the study. His/her reference to the study being underpowered seems to refer to the fact that 11% of the patients with melioidosis had an active cancer. However, almost none of these patients were taking TMP-SMX at the time that their melioidosis was diagnosed. In fact, almost all of them – who survived long enough – received TMP-SMX treatment as eradication therapy for their melioidosis. The cohort of 446 patients with confirmed melioidosis was not the cohort that we used to examine the utility of TMP-SMX prophylaxis.

We examined the utility of TMP-SMX prophylaxis in the 4536 patients with cancer who received anti-cancer therapy, 652 of whom received TMP-SMX prophylaxis for *Pneumocystis jirovecii.* We were able to compare the incidence of melioidosis in the patients who did – and did not – receive TMP-SMX prophylaxis in this cohort to determine the NNT to prevent 1 episode of melioidosis. We would argue that if 652 individuals in a cohort of 4536 patients are taking TMP-SMX prophylaxis, we are not underpowered to answer our study question.

The aim of this part of the study was to determine whether there was any benefit of TMP-SMX chemoprophylaxis for melioidosis in patients with cancer receiving chemotherapy in the FNQ region, and whether we should change our practice and prescribe it. The very low incidence of melioidosis in patients receiving anti-cancer therapy in our region, the risk of side effects of TMP-SMX and the potential for deleterious effects on local antimicrobial resistance mean that our data do not presently support its routine prescription in this region of Australia.
