# Supplementary material for: The characteristics and clinical course of patients with melioidosis and cancer
Source: PLoS Negl Trop Dis. 2024 Oct 25;18(10):e0012631. doi: 10.1371/journal.pntd.0012631 (PMC11540213; doi:10.1371/journal.pntd.0012631)
Supplement: Attachment — Submitted filename: PNTD-D-24-00958R1_Response to Editorial comments.docx [file pntd.0012631.s005.docx]

Response to additional Editorial comments and queries

We thank the Editorial staff for all the time that they have taken to review the revised version of our manuscript and the very helpful comments that they have made to improve the work. Please find below our point-by-point responses (in black) to their comments (in blue).

In reviewing our data to address their queries, we have identified some errors in the presentation of the data in the tables. We have amended these errors – which do not change the conclusions of the study – in the revised submission.

These errors were

1. In table 2, 1 patient was presented twice, and another was missing. This has been rectified. This has no impact on our study’s conclusions.
2. There were 16 – not 17 – patients who developed melioidosis after receiving anti-cancer therapy without receiving TMP-SMX. This error was caused by the double counting of the patient in table 2 that we highlighted. This has no impact on our study’s conclusions.
3. In the initial submission, we misreported the number of deaths due to melioidosis among the patients receiving anti-cancer therapy. While the total number of deaths was correct, some of these deaths were due to the patient’s cancer, not their melioidosis, an important distinction. We reported that the patient who developed melioidosis after having received TMP-SMX died due to melioidosis. This was not the case. He died from his malignancy. Furthermore, only 3 – not 6 – of the patients who not receiving TMP-SMX who developed melioidosis, died from their melioidosis. Again, these 3 mis-classified deaths were due to their malignancy. We have represented these data in table 4 and we believe that this error does not have any impact on the conclusions of our study.
4. We identified a greater number of patients receiving anti-cancer therapy during the study period; this changes the denominators in table 4 but does not change the conclusions of the study.

Therefore, overall, only 3/17 (18%) of individuals who developed melioidosis after receiving anti-cancer therapy died from their melioidosis. All three deaths occurred in patients who had not received TMP/SMX prophylaxis for *P. jirovecii* pneumonia. Previously we had suggested that there was no difference in the incidence of fatal melioidosis between patients receiving – and not receiving – TMP/SMX chemoprophylaxis. With our revised data, there was an absolute difference in the proportion of individuals who had fatal melioidosis between patients who did – and did not – receive TMP-SMX prophylaxis, but this difference did not meet statistical significance (0/737 (0%) versus 3/4031 (0.07%), p=0.60). We have revised table 4 accordingly.

This does not change the main conclusion of the paper that melioidosis is an extremely rare complication of anti-cancer therapy in FNQ. And presently, as we outline in the discussion, the potential adverse effects of TMP-SMX outweigh the putative benefits of TMP-SMX preventing melioidosis in the region

Editorial comment:

Thank you very much for responding in detail to the reviewers' concerns. While much of the manuscript has been improved, the final section focused on TMP-SMX treatment and development of melioidosis among patients with cancer remains problematic for the following reasons:
- As I understand it, this part of the analysis was conducted on patients at Cairns Hospital that were identified using the MOSAIQ programme. I suspect that these data were then integrated with the hospital laboratory culture data but I do not believe that this is explicitly stated. Please update the methods and the appropriate section of the results (starting at line 306) to ensure that readers understand both this strategy and that this is a separate parent cohort from the earlier analyses.

**Response**: This was, indeed, the case. We have amended the text accordingly (lines 215-218).

- In this analysis, is it correct to assume is that most patients who were treated for cancer at Cairns Hospital are likely to have melioidosis diagnosed at the same hospital?

**Response**: Yes. Cairns Hospital is the sole tertiary hospital for the Far North Queensland region. All myelosuppressive chemotherapy and anti-cancer immunotherapy administered to FNQ residents is prescribed by clinicians at the Cairns Hospital. Melioidosis is a notifiable disease in the state of Queensland and all cases of melioidosis are diagnosed in the Cairns Hospital laboratory, which is the referral laboratory for the region. We have added text to the methods to make this clearer (lines 174-176). There are 3 smaller laboratories in the region – that are based in district hospitals – which do the initial processing of microbiological specimens, but all confirmation of *B. pseudomallei* infection takes place in the Cairns Laboratory.

Any individual diagnosed with melioidosis – who is not already an inpatient at the Cairns Hospital – would be discussed with the Department of Infectious Diseases at Cairns Hospital. Over 90% of the patients with a diagnosis of melioidosis will ultimately be admitted to Cairns Hospital, given the challenges of managing this potentially life-threatening infection in smaller hospitals with less critical care support.

In this study there were only 2 individuals with a malignancy and confirmed melioidosis who were not admitted to Cairns Hospital. Both were admitted to district hospitals in the region – and the laboratory specimens which isolated *B. pseudomallei* were transferred to the Cairns Hospital laboratory where the microbiological diagnosis of *B. pseudomallei* was confirmed. In one – a patient with metastatic liver cancer – palliative care was initiated in his district hospital due to his poor prognosis from his underlying malignancy. The other, who had significant comorbidity, was managed in his district hospital – 800km from Cairns – before transfer to a residential aged care facility. The management of his melioidosis was supervised remotely by the Infectious Diseases service at Cairns Hospital.

- In order to appropriately evaluate the relative incidence of melioidosis in this cohort in those prescribed TMP-SMX vs not, (differential) follow up time is an essential consideration, yet does not seem to have been addressed. For example, if patients receiving TMP-SMX were followed up, on average, for longer time periods than patients not receiving TMP-SMX, conceivably more cases of melioidosis may be identified in those receiving TMP-SMX. Alternatively, if more patients receiving TMP-SMX died (due to their cancer or for reasons unrelated to melioidosis), then they would no longer be at risk for melioidosis and TMP-SMX may appear to be associated with lower rates of melioidosis.

**Response**: We thank the Editors for raising this point, but we don’t feel that any differences in follow up time between the patients receiving – and not receiving – TMP-SMX prophylaxis would explain our findings. We are unsure why patients receiving TMP-SMX during their anti-cancer therapy would be followed up, systematically, for longer – or shorter – periods of time than those who did not. It is important to note that the prescription of TMP-SMX in the cohort was not discretionary. Prescription of TMP/SMX was part of the chemotherapy protocol to reduce the risk of *Pneumocystis jirovecii* pneumonia in individuals receiving specific anti-cancer therapy regimens (as we highlight in lines 213-218)

So other patient characteristics (that might be associated with closer follow up) did not influence its prescription. There was also no change in the proportion of individuals receiving TMP/SMX over the study period (p for trend=0.12) (see below table) supporting the contention that there was no systematic difference in the follow up time between the two groups.

Table. Number and proportion of patients receiving TMP/SMX during their anti-cancer therapy during the study period.

| Year | No TMP/SMX | Received TMP/SMX | % receiving TMP/SMX |
| --- | --- | --- | --- |
| 2008 | 54 | 12 | 18.2 |
| 2009 | 170 | 31 | 15.4 |
| 2010 | 160 | 27 | 14.4 |
| 2011 | 266 | 48 | 15.3 |
| 2012 | 247 | 41 | 14.2 |
| 2013 | 491 | 86 | 14.9 |
| 2014 | 294 | 46 | 13.5 |
| 2015 | 277 | 51 | 15.5 |
| 2016 | 327 | 50 | 13.3 |
| 2017 | 268 | 53 | 16.5 |
| 2018 | 298 | 52 | 14.9 |
| 2019 | 259 | 51 | 16.5 |
| 2020 | 280 | 47 | 14.4 |
| 2021 | 252 | 52 | 17.1 |
| 2022 | 265 | 67 | 20.2 |
| 2023 | 123 | 23 | 15.8 |

While patients having higher intensity anti-cancer therapies were more likely to receive TMP-SMX and would, in general, have more aggressive malignancies with a poorer short-term prognosis, there was no statistical difference in the development of melioidosis amongst those having high intensity chemotherapy who did – and those who did not – receive TMP/SMX (1/227 (0.44%) v 2/158 (1.27%) relative risk: 0.35 (0.03-3.81) p=0.37). Indeed, there was no statistical difference in the risk of developing melioidosis between those taking and those not receiving TMP-SMX at any intensity of anti-cancer therapy.

It is also important to remember that most of the patients will have had several years of follow up. Even excluding non-melanomatous skin cancers, the five-year survival of patients with a diagnosis of cancer in North Queensland is 69% (https://cancerqld.org.au/content/uploads/cancer-fact/Regional-cancer-statistics_NQ.html). And even in those individuals who are not cured of their cancer, their cancer is not a strong independent risk factor for melioidosis in the absence of anti-cancer therapy. As we note in the paper only 10 of the 446 patients with complete data who developed melioidosis between 1998 and 2023 in the FNQ region had cancer, no other risk factors for melioidosis and did not receive myelosuppressive anti-cancer therapy.

Melioidosis was a very rare complication of anti-cancer therapy in our cohort: there were only 17 cases of melioidosis in 4768 patients receiving anti-cancer therapy. It seems unlikely that this finding is due to short follow up. We documented whether the patients with cancer and melioidosis had received any anti-cancer therapy in the prior 12 months, a deliberately generous period that would tend to overestimate of the time that anti-cancer therapy may have resulted in immunosuppression and increase the risk of melioidosis. It is also important to note that many of the 4768 individuals would have received multiple lines of anti-cancer therapy, so in fact the risk per course of anti-cancer therapy would be even lower than the rate of 0.36% that we have documented. Also 7/17 (41%) had other risk factors for melioidosis (including chronic lung disease, diabetes, hazardous alcohol use) so it may be that anti-cancer therapy played a limited role in the development of melioidosis in these cases.

Finally, the duration of follow up may be less important than the Editors suggest as most anti-cancer therapies would be expected to increase the risk of melioidosis for a relatively short period of time. The risk of melioidosis would be greatest during the time that the patient was receiving anti-cancer therapy or very shortly afterwards. Once the anti-cancer therapy is finished there will be immune recovery with the risk of melioidosis decreasing accordingly. For example, if a patient receives adjuvant chemotherapy for their resected bowel cancer or breast cancer (the two most common cancers in women in FNQ) immune recovery will occur shortly after they complete their chemotherapy and, all things being equal, they will not be at any ongoing increased risk of melioidosis.

Similarly, if the impact of TMP-SMX chemoprophylaxis on the incidence of melioidosis would also be expected to be short lived. Once it is ceased, there would no longer be any protective effect. The dose of TMP-SMX chemoprophylaxis for *P. jirovecii* pneumonia (160/800 bd on two days in a week during the anti-cancer therapy) would not be expected to treat latent melioidosis (eradication dose 320/1600 bd every day for ≥3 months) and would also not prevent future episodes of new infection.

- I would caution the authors about using the phrase "impact of TMP-SMX" (or similar phrasing) when any relationship between TMP-SMX and melioidosis in this observational cohort can at best be considered a correlation, not causation.

**Response**: We have amended the text, replacing “impact of TMP-SMX” with “Association between TMP-SMX prophylaxis during anti-cancer therapy and the subsequent diagnosis of melioidosis.” We would be happy to substitute another phrase if the Editors had a specific suggestion that they would prefer.

- Confounders should also be considered in fully evaluating whether TMP-SMX is associated with altered incidence of melioidosis. For example, if TMP-SMX is preferentially prescribed to patients who are younger and have fewer comorbidities, then any reduced incidence of melioidosis may be related to the baseline characteristics of patients rather than TMP-SMX treatment. I recognize that the very low numbers of patients developing melioidosis (1 in the TMP-SMX group and 17 in the no-TMP-SMX group, as I read Table 4) essentially precludes any meaningful multivariable analysis. However, at the very least I would suggest including a table that describes the baseline characteristics of both groups to permit a qualitative evaluation by readers.

**Response**: We thank the Editors for raising this issue. However, the prescription of TMP-SMX was not discretionary. Prescription of TMP/SMX was part of the chemotherapy protocol to reduce the risk of *Pneumocystis jirovecii* pneumonia in individuals receiving specific anti-cancer therapy regimens. It was not therefore prescribed, preferentially – as the Editors suggest – to younger patients or those with fewer comorbidities. If a patient had an absolute contraindication to TMP/SMX (for instance hypersensitivity) they received a second line agent such as dapsone. We have revised the methods to make this clearer (lines 213-218).

We have provided a table to compare the baseline characteristics of three groups of patients with an active cancer and a diagnosis of melioidosis (supplementary table 3).

1. The 30 patients who received no anti-cancer therapy prior to their diagnosis of melioidosis
2. The 1 patient who received TMP/SMX prophylaxis prior to their anti-cancer therapy.
3. The 16 patients who received no TMP/SMX prophylaxis prior to their anti-cancer therapy.

As the Editors suggest it is not possible to perform any meaningful statistical analysis of the three groups given the small numbers, however, we hope that this table and the tables 2 & 3 (which describe the age, cancer and some of the comorbidities of the individuals diagnosed with melioidosis) will allow the reader to evaluate the cohort qualitatively.

- The calculation of NNT is well-intentioned but, in my view, fraught with challenges when applied in this context. Please see Stang A, et al, J Clin Epidemiology 63 (2010) 820-25, for a summary of the issues. I would strongly consider eliminating NNT. Instead, I think that bolstering the analysis of melioidosis incidence among those receiving TMP-SMX vs not, as suggested above, will provide readers and clinicians with sufficient relevant data.

**Response**: We thank the Editors for raising this point and providing a helpful reference. While the NNT is intuitively useful for clinicians, we agree that there are challenges with applying it to our cohort. We have amended the presentation of our data accordingly, deleting any reference to NNT and instead we have presented the relative risk (with appropriate 95% confidence intervals) which we feel is the better way to present the data. We have calculated statistical significance using a one-sided Fisher’s exact test which would tend to increase the likelihood of identifying a statistically significant association.
